# Impact of oral health on health-related quality of life: a cross-sectional study

**DOI:** 10.1186/s12903-016-0211-2

**Published:** 2016-05-12

**Authors:** Miriane Lucindo Zucoloto, João Maroco, Juliana Alvares Duarte Bonini Campos

**Affiliations:** Faculdade de Odontologia de Araraquara, Universidade Estadual Paulista “Júlio de Mesquita Filho”, Rua Humaitá, 1680. Centro, 14801-903 Araraquara, São Paulo Brazil; Instituto Universitário de Ciências Psicológicas, Sociais e da Vida - ISPA, Rua Jardim do Tabaco n° 34, 1149-041 Lisboa, Portugal

**Keywords:** Oral health, General health, Quality of life, Oral health surveys

## Abstract

**Background:**

Despite the consensus regarding the existence of a relationship between “impacts on oral health” and “health-related quality of life”, this relationship, considering the latent nature of these variables, is still poorly investigated. Thus, we performed this study in order to determine the magnitude of the impacts of oral health, demographic and symptom/clinical variables on the health-related quality of life in a Brazilian sample of dental patients.

**Methods:**

A total of 1,007 adult subjects enrolled in the School of Dentistry of São Paulo State University (UNESP) - Araraquara Campus for dentistry care between September/2012 and April/2013, participated. 72.4 % were female. The mean age was 45.7 (SD = 12.5) years. The Oral Health Impact Profile (OHIP-14) and the Short Form Health Survey (SF-36) were used. The demographic and symptom/clinical variables collected were gender, age, economic status, presence of pain and chronic disease. The impact of studied variables on health-related quality of life were evaluated with a structural equation model, considering the factor “Health” as the central construct. The fit of the model was first analyzed by the evaluation of the goodness of fit indices (χ^2^/df ≤ 2.0, CFI and TLI ≥ 0.90 and RMSEA < 0.10) and the evaluation of the variables’ impact over health-related quality of life was based on the statistical significance of causal paths (β), evaluated by z tests, for a significance level of 5 %.

**Results:**

We observed adequate fit of the model to the data (χ^2^/df = 3.55; CFI = 0.95; TLI = 0.94; RMSEA = 0.05). The impacts on oral health explained 28.0 % of the variability of the health-related quality of life construct, while the total variance explained of the model was 39.0 %. For the demographic and symptom/clinical variables, only age, presence of pain and chronic disease showed significant impacts (*p* < 0.05).

**Conclusion:**

The oral health, age, presence of pain and chronic disease of individuals had significant influence on health-related quality of life.

## Background

The health-related quality of life is a complex and multidimensional construct composed of a set of concepts. The first articles that made reference to the term “health-related quality of life” were published in the mid-1980s and since then, many changes have been attributed to this definition and measurement [1]. According to Dijkers [2], health-related quality of life is an important component of quality of life, being composed by physical, cognitive, emotional and social aspects. Nowadays, it is well known that it can be modulated directly or indirectly by imbalances in health as diseases, disorders or injuries, being sensitive to the signs, symptoms and treatment effects [3]. Thus, this construct can be assessed both by general or by specific approaches, such as oral health [4].

In the literature, there are several theoretical approaches and conceptual frameworks proposed to assess the Health and the Oral health-related quality of life [4–9]. Individual perceptions regarding the oral health impact profile have been growing in importance since they can influence the self-care practices and can have a direct effect on health-related quality of life of individuals.

One of the most widely used instrument to assess the “impact on the oral health” is the Oral Health Impact Profile (OHIP) proposed by Slade and Spencer [8] based on the model proposed by Locker [10]. The OHIP evaluates three conceptual domains (physical, psychological and social) that quantify the individual perception of the impacts generated by oral problems in general health.

The health-related quality of life, in turn, has been evaluated by estimating the impact of symptoms, disabilities or limitations that may result in disturbance of individual well-being [11]. The Short Form Health Survey (SF-36) is a generic health indicator most commonly used for this purpose [11]. The SF-36 consists of two conceptual domains “physical health” and “mental health”, which converge to the individual perception of “health”, and the estimation of health-related quality of life of individuals. In this instrument eight health concepts were measured, selected from dozens included in the Medical Outcomes Study model [12].

According to Locker studies [7] oral health can affects people physically and psychologically and can influence many aspects as how they enjoy life, speak, chew, taste food, socialize and the social well-being. Thus, some recent studies in the scientific literature evaluated the impact of general [13] and specific aspects of oral health, as the use of prostheses [14], surgical treatments [15], parafunctional habits [16], dental pain [17], among others, on quality of life in different samples, being common the association of oral conditions evaluated on the factors of health-related quality of life of patients.

Despite the consensus regarding the existence of a relationship between “impacts on oral health” and “health-related quality of life” [7] and the development of some models to evaluate these constructs [18, 19], this relationship, considering the latent and multidimensional nature of variables, is still poorly investigated. As far as we know, only the study of Reissman et al. [18] aimed at evaluating the contribution of oral health impacts, demographic and symptom/clinical variables on health-related quality of life, preserving the latent nature of these variables. However, the theoretical model presented by these authors considers both the health-related quality of life and the oral health impact profile as dependent variables and estimates the relationship between them through correlational analysis.

Another aspect to be considered is that, through the assessment of the impact of oral problems on health-related quality of life, we can make a vital contribution to improve the prevention and dental intervention strategies, promoting a better quality of life for individuals.

Thus, we performed this study in order to determine the magnitude of the impacts of oral health, demographic and clinical variables on the health-related quality of life in a Brazilian sample of dental patients.

## Methods

### Study design and sampling

A cross-sectional study with non-probabilistic sampling design was developed.

The estimation of the sample size was performed considering the proposal of Hair et al. [20]. According to this author, the sample size necessary was estimated considering the need from 7 to 10 subjects per parameter to be estimated in the model. (final model’ parameters: OHIP: *n* = 34, SF-36: *n* = 77, demographic and symptom/clinical variables: *n* = 5). Thus, we obtained an estimate of the sample size of 812 to 1,160 subjects [20]. Assuming a loss rate of 15 %, the minimum sample size required for structural equation modelling was estimated at 956 to 1,365 subjects.

A total of 1,925 adult patients, who sought dental care in the School of Dentistry of São Paulo State University (UNESP), Araraquara Campus from September of 2012 to April of 2013 were invited to participate. Of these, 1,203 agreed to participate (adhesion rate = 62.5 %). Only those participants who completed all items of the demographic questionnaire and measuring instruments (SF-36 and OHIP-14) were included, resulting in a final sample size of 1,007 patients.

### Study variables

The study variables were gender, age, economic class, presence of pain at the time of questionnaire application (yes or no) and presence of chronic disease (yes or no). The choice of these variables were based in previous studies and models of oral and general health assessment [18, 21–23]. To identify patients with pain, all the participants responded the question “Are you in pain at this moment?” If yes, they indicated the location. It should be clarified that it was not carried out any clinical examination to verify the presence or absence of pain, i.e., for this study was considered only the referred pain. The economic classes were classified according to the Brazilian Economic Classification Criterion - ABEP [24]. To characterize the sample, other information were collected like the type of chronic disease and the dental status (dentate, edentulous or partial edentulous), use of dental prosthesis (yes or no) and the type (fixed partial denture, removable partial denture or complete denture).

### Measuring instrument

The oral health impact profile was estimated using the Portuguese reduced version of the Oral Health Impact Profile (OHIP-14) proposed by Oliveira and Nadanovsky [25]. This version of the instrument is composed by 14 items arranged in seven first-order factors (functional limitation, physical pain, psychological discomfort, physical disability, psychological disability, social disability and handicap). The answers are given in a five-point type Likert scale (0 = never, 1 = rarely, 2 = sometimes, 3 = often, 4 = always). Zucoloto et al. [26] evaluated the psychometric properties of this instrument in the sample of this study and they attested the validity and reliability of the OHIP-14. They proposed a third-order hierarchical model composed by the second-order factors “Physical”, “Psychological” and “Social” and one third-order factor called “OHIP”. The fit of third-order hierarchical model was adequate (λ = 0.62-0.83, χ^2^/df = 7.67, CFI = 0.94, GFI = 0.93 and RMSEA = 0.08; Cronbach’s alpha = 0.62-0.77; Composite Reliability = 0.63-0.77) and this was the model used in this study.

The Health-related quality of life was estimated using the Portuguese version of the Short Form Health Survey (SF-36), in the standard format (recall period of 4 weeks), provided by Quality-Metric Incorporation® (Copyright: QM13691). The factorial structure used was composed of seven first-order factors (physical functioning, role-physical, bodily pain, general health, social functioning, role-emotional and mental health and well-being), two second-order factors (Physical Health and Mental Health) and a third-order factor (Health), whose psychometric properties have been attested in a previous study for the sample of this study (λ = 0.46–0.90, χ^2^/df = 5.90, CFI = 0.90, TLI = 0.90 and RMSEA = 0.06; Cronbach’s alpha = 0.76–0.93; Composite Reliability = 0.70–0.94) [27]. The answers are given in a type Likert scale of three point for the factor “Physical functioning” and five points for the other factors.

### Procedures

The questionnaires were self-completed in the waiting room of the clinics of the School of Dentistry of São Paulo State University (UNESP) (general clinic and specialties clinics such as prosthesis, endodontics, surgery and periodontics), before the dental procedure. Patients who had difficulty in filling the questionnaires were interviewed (16.8 %). The questionnaires were presented in random order to minimize the bias in the fill. Only individuals aged 18 years or more participated.

### Statistical Analysis

#### Structural Model

A structural equation model was built considering the demographic, symptom/clinical and OHIP variables’ impact on the 3rd order hierarchical factor “Health”, estimated by the SF-36, (dependent variable). The variables “oral health impact profile”, age, gender, economic class, presence of pain and chronic disease were considered, in the model, as predictors variables. The goodness of fit of this causal model was evaluated on the polychoric correlation matrix using Weighed Least Squares Mean and Variance Adjusted (WLSMV) estimation, as implemented in the software MPLUS 6.0 (Muthén & Muthén, Los Angeles, CA). The fit of the model was first analyzed by the indices of goodness of fit and was considered adequate if χ^2^/df ≤ 2.0, CFI e TLI ≥ 0.90 and RMSEA < 0.10 [28]. The significance of the predictor variables on the Health central construct was judged form the statistical significance of the paths (β), assessed by z tests, for a significance level of 5 % [28].

### Ethical aspects

This study was authorized by the Research Ethics Committee of the Faculty of Dentistry of Araraquara - UNESP (CAAE: 01040312.5.0000.5416-n° 50802). The study included only individuals over 18 years of age who agreed and signed the Free and Informed Consent Form. Authorizations for the use of the instruments were acquired together with the authors (OHIP-14) and competent agencies (SF-36 Quality Metric Inc. - License: QM13691).

## Results

The mean age of the participants was 45.7 (SD = 12.5) years and 72.4 % were female.

Table 1 shows the distribution of the patients according to demographic and symptom/clinical characteristics.

**Table 1:** Distribution of patients according to demographic and symptom/clinical characteristics. Araraquara, São Paulo, Brazil, 2013

Characteristics	n (%)
Economic Class (Monthly income)	
A (US$2.017,80-US$3.383,11)	31 (3.1)
B (US$724,55-US$1.217,28)	319 (31.7)
C (US$402,43)	533 (52.9)
D ou E (US$89,86 a US$184,07)	124 (12.3)
Chronic desease	
No	752 (74.7)
Yes	255 (25.3)
Pain	
No	649 (64.4)
Yes	358 (35.6)
Dental condition	
Dentate	361 (35.8)
Partial edentulous	583 (57.9)
Edentulous	63 (6.3)
Dental prosthesis	
No	605 (60.1)
Yes	402 (39.9)
Type of dental prosthesis	
Fixed partial denture	171 (42.5)
Pacial removable denture	133 (33.1)
Complete denture	98 (24.4)

The sample consisted mostly by partial edentulous subjects with low economic status. Of participants who reported the presence of pain (*n* = 358), 61.2 % had a toothache, 17,3 % a face pain, 9,8 % a head pain, 7,8 % a pain in the ear region/temporomandibular joint and 3,9 % in other orofacial region. The most prevalent chronic diseases were hypertension (25.3 %) and diabetes mellitus (20.2 %).

The structural model composed by all independente variables presented adequate fit to the sample (χ^2^/df = 3.55; CFI = 0.95; TLI = 0.94; RMSEA = 0.05-Explained variance of the model: 39.0 %). OHIP has negative and significant impact over health-related quality of life (β = −0,53; *p* < 0,05). The higher the impact on oral health, the lower is the health-related quality of life. This path explains 28.0 % for the variability of central construct. Regarding demographic and symptom/clinical variables, the age, the pain and the presence of chronic disease showed negative and significant trajectories (*p* < 0,05), although they presented low contribution to the explanation of the central construct, being 3.6, 1.2 and 4.4 %, respectively. There was no significant contribution (*p* > 0.05) of gender or economic class to health-related quality of life.

**Fig. 1:**
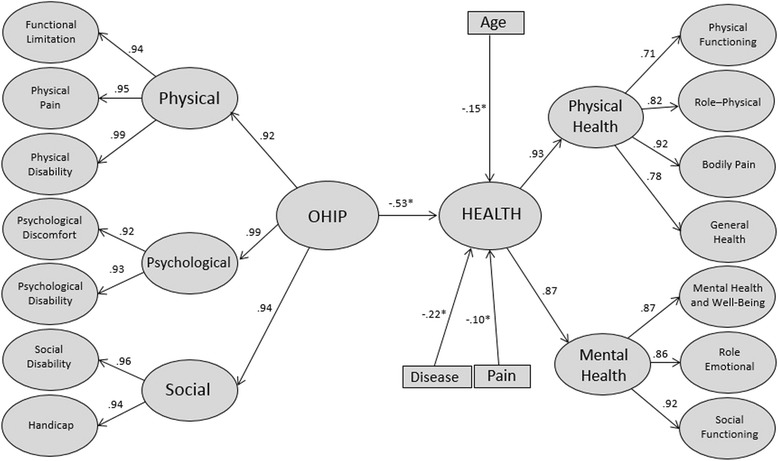
Structural refined model for assessment the contribution of the impact of oral health (OHIP), demographic and symptom/clinical variables on Health-related Quality of Life (Health). Araraquara, São Paulo, Brazil, 2013

Subsequently, a refined model (including only significant variables) was analysed. The refined model also presented an adequate fit to the data (β = −0.53 to −0.10, χ^2^/df = 4.27, CFI = 0.93, TLI = 0.93, RMSEA = 0.06) (Fig. 1). The explained variance of the refined model was 36.1 % and the contribution of the OHIP to the central construct continued on 28.0 %.

## Discussion

The results presented in this study show a significant contribution of the impact of oral health on health-related quality of life, suggesting to further consider this construct in health-related quality of life research [4, 7].

In this present study, we introduce a distinct theoretical proposal, aiming at estimating the contribution of the impact of oral health on health-related quality of life. This proposal preserves the latent characteristic of the variables (OHIP-14 and SF-36) and the functional relationship of each to the construction of the concepts, ie, the oral health impact profile were considered the independent variable and the health-related quality of life the dependent variable. Thus, we presented a possibility of develop a predictive model that goes beyond correlational patterns between the variables.

It is worth mentioning that is not possible to directly compare our results with previous published research, because there is a lack of studies that preserve the latent characteristics of the constructs assessed. The theoretical model presented is different of others previously tested in the scientific research.

Another noteworthy aspect is that, despite the widespread use of the SF-36 and OHIP-14 in the literature, few studies investigated the psychometric properties of the instruments prior to use in their study samples [5, 29]. This step was presented in this study and it is essential to provide evidence regarding the quality (validity and reliability) of the obtained data [28, 30, 31].

In the predictive model proposed (Fig. 1), it can be observed a significant inverse relationship of the impacts of oral health on health-related quality of life. This relationship accounts for 28.0 % of the variability of the central construct, which can be considered an important contribution, in view of the complexity and multidimensionality of the health-related quality of life. This fact signals to the importance of oral health to general health of individuals. Therefore, our results emphasize the importance of the oral health, which should demand special attention by health professionals [7]. It is noteworthy, however, that the important explained variance detected in the structural model evaluated can be related to the characteristics of the sample. The study included only patients who sought dental care, it means, they have some impairment/dissatisfaction with their oral health. Dental patients potentially have worst dental clinical status, more perceived dental treatment need and more impact of oral health on general health. Thus, further studies should test this model to be tested in populations without oral problems to verify the contribution from the impact caused by oral problems in these persons.

Among the demographic variables included in the model, only age was a significant predictor (Fig. 1), i.e., the health-related quality of life is worse with increasing age. Similar results were found in published research. This results can be justified by greater impact of oral and systemic diseases in elderly individuals [19, 32–35].

Regarding symptom/clinical variables, the presence of “chronic disease” and “pain” showed significant contributions to the health-related quality of life. Importantly, the presence of pain and chronic disease have been considered essential variables in studies of health-related quality of life and oral health-related quality of life due to impacts on the wellbeing of individuals [7, 36].

A limitation of this study may be the non-probabilistic sampling design adopted. However, this strategy has been commonly utilized in validation studies. The use of sufficient sample size ensures credibility of the decision-making that results from the statistical tests. Another limitation is the use of self-referred information about dental condition and use/type of dental prosthesis which limited their inclusion in the predictive model. This type of data collection strategy was used due to the large sample size. However, it is suggested the fulfillment of studies that include oral clinical examination as a methodological strategy in order to verify the possible contribution of these variables to the model presented.

With this study, we provide relevant information to healthcare professionals, on the impacts caused by oral problems in health-related quality of life. This knowledge can guide the development of preventive strategies and more resolute and extensive treatments focusing not only in solving oral problems, but also considering their impact on the overall health of individuals.

## Conclusion

The variables “oral health impact profile”, age, chronic disease and presence of pain showed significant influences on health-related quality of life.

## Abbreviations

CFI: comparative fit index
OHIP: oral health impact profile
OHIP-14: oral health impact profile-short form
RMSEA: root mean square error of approximation
SF-36: short form health survey
TLI: Tucker-Lewis index
WLSMV: weighed least squares mean and variance adjusted
β: regression coefficient
χ^2^/df: ratio of chi-square by the degrees of freedom
